# Genome-Wide Identification and Expression Profiling of CBL-CIPK Gene Family in Pineapple (*Ananas comosus*) and the Role of *Ac*CBL1 in Abiotic and Biotic Stress Response

**DOI:** 10.3390/biom9070293

**Published:** 2019-07-20

**Authors:** Mohammad Aslam, Beenish Fakher, Bello Hassan Jakada, Lihua Zhao, Shijiang Cao, Yan Cheng, Yuan Qin

**Affiliations:** 1 Key Laboratory of Genetics, Breeding and Multiple Utilization of Crops, Ministry of Education, Fujian Provincial Key Laboratory of Haixia Applied Plant Systems Biology, College of Crop Science, Fujian Agriculture and Forestry University, Fuzhou 350002, Fujian, China; 2 State Key Laboratory of Ecological Pest Control for Fujian and Taiwan Crops, College of Plant Protection, Fujian Agriculture and Forestry University, Fuzhou 350002, China; 3 Life Science College, Fujian Agriculture and Forestry University, Fuzhou 350002, Fujian, China; 4 College of Forestry, Fujian Agriculture and Forestry University, Fuzhou 350002, Fujian, China; 5 State Key Laboratory for Conservation and Utilization of Subtropical Agro-Bioresources, Guangxi Key Lab of Sugarcane Biology, College of Agriculture, Guangxi University, Nanning 530004, Guangxi, China

**Keywords:** CBL-CIPK, pineapple, genome-wide, salt tolerance, biotic stress

## Abstract

Ca^2+^ serves as a ubiquitous second messenger regulating several aspects of plant growth and development. A group of unique calcium sensor proteins, calcineurin B-like (CBL), interact with CBL-interacting protein kinases (CIPKs) to decode the Ca^2+^ signature inside the cell. Although CBL-CIPK signaling toolkit has been shown to play significant roles in the responses to numerous stresses in different plants, the information about pineapple CBL-CIPK remains obscure. In the present study, a total of eight *Ac*CBL and 21 *Ac*CIPK genes were identified genome-wide in pineapple. The identified genes were renamed on the basis of gene ID in ascending order and phylogenetic analysis divided into five groups. Transcriptomic data analysis showed that *Ac*CBL and *Ac*CIPK genes were expressed differentially in different tissues. Further, the expression analysis of *Ac*CBL1 in different tissues showed significant changes under various abiotic stimuli. Additionally, the ectopic expression of *Ac*CBL1 in *Arabidopsis* resulted in enhanced tolerance to salinity, osmotic, and fungal stress. The present study revealed the crucial contribution of the CBL-CIPK gene in various biological and physiological processes in pineapple.

## 1. Introduction

In living organisms, the precise perception and timely decoding of environmental and developmental signals are essential for survival. The signal transduction pathway involved in this process includes several indispensable components including calcium (Ca^2+^), which serves as a ubiquitous second messenger in all eukaryotes [1,2,3]. Various organelles such as endoplasmic reticulum (ER), mitochondria, and vacuoles work as Ca^2+^ store and help in the maintenance of a critical balance of Ca^2+^ inside the cell [4]. Acknowledging the stress or developmental signals, the cytosolic calcium concentration rapidly shoots up as an early response [5]. Consistently, in plants, the rapid increase of cytosolic Ca^2+^ concentration is well documented against several stress factors such as salinity, drought, and cold [3,6,7]. In addition, pH dynamics, pathogens, phytohormones, light signal, and drugs, as well as the development of a pollen tube and regulation of guard cells are also linked with changes in the concentration of Ca^2+^ [8,9,10,11,12,13]. The elevated Ca^2+^ level inside the cell is then recognized by several calcium-binding proteins or calcium sensors [5]. After sensing the elevation in Ca^2+^ level these calcium sensors bind to calcium in their specific motifs (such as EF-hands), which changes their phosphorylation status. The change in the phosphorylation status of calcium sensors activates several protein kinases which sometimes lead to a protein phosphorylation cascade [14]. A number of calcium sensors have been reported in plants including calmodulin (CaM), calcineurin B-like (CBLs), and calcium-dependent protein kinases (CDPKs) [15].

CBLs are plant-specific genes that encode a protein similar to the calcineurin B subunit of protein phosphatase of yeast and animal cells [16]. CBLs were initially identified in *Arabidopsis* as an essential player of the salt stress response [17]. CBLs specifically bind with the conserved C-terminal NAF/FISL domain of CBL-interacting protein kinases (CIPKs) [18]. Both, the CBLs and CIPKs are encoded by the multigene family in plants. The *Arabidopsis* genome encodes 10 members of CBLs and 26 members of CIPKs [19]. In addition, individual CBLs can physically interact with multiple CIPKs after sensing the specific calcium signature of a specific signal in the cellular environment [20]. This interaction activates the downstream components of the CBL-CIPK signaling pathway, including ion channels and transporters involved in plant physiological and developmental response [18,21]. The complex signaling network of CBL-CIPK in plants participates in several essential functions of plant growth and development. Calcineurin B-like proteins have been shown to play key roles in biotic and abiotic stress responses [3]. For example, the CBL7 mediates the alkaline stress response in *Arabidopsis* by promoting the self-inhibition of plasma membrane H+-ATPase [22]. Consistently, CBL1 plays key roles in cold stress [23,24], aluminum stress [25], activation of AKT1 channel [26], glucose, and gibberellic acid signal [27]. In addition, CBL1 along with CBL9, is also involved in pollen germination and pollen tube growth [28], and in the regulation of potassium transport [29]. Similarly, CBL2 and CBL3 are involved in seed development and seed morphology [30], CBL3 in potassium homeostasis [31], and CBL4-CIPK6 complex modulates the activity and plasma membrane targeting of the K^+^ channel AKT2 [32].

Abiotic stresses greatly limit plant growth and development. Among them, salt stress is a prime abiotic stress which severely affects the plant productivity and significantly decreases the agricultural yield. Roughly, 20% of the irrigated agricultural land is under salt stress [33]. In plants, salt stress initiates the highly conserved pathways which export Na^+^ ions outside the cell, known as salt overlay sensitive (SOS) pathway [17,33]. SOS pathway key players SOS2 kinase and SOS1 Na^+^ antiporter are activated within 2 h of salt stress [34]. In *Arabidopsis*, CBL10 reads and decodes the salt-induced cytosolic changes in calcium concentration and further activates the CIPK24 (SOS2 kinase) [3,17,35]. Salinity induced SOS2 kinase then phosphorylates plasma membrane Na^+^/H^+^-ATPase (SOS1), which transports the Na^+^ to apoplast from the cytoplasm [34,35].

Pineapple, *Ananas comosus* (L.) Merr., belonging to the family Bromeliaceae is grown commercially in tropical and subtropical regions of the world. Pineapple is highly appreciated for its delicious fruit and aroma, ranking it among the top three economically important tropical fruits in the world, with banana and citrus [36]. The growth and development of the pineapple plant is seriously affected by the biotic and abiotic stresses. However, it can withstand up to some level of drought conditions owing to its morphological features and plants crassulacean acid metabolism [37]. Due to its worldwide cultivation and immense economic and research significance, researchers have shown considerable interest in fishing out crucial functional genes from pineapple.

CBL-CIPK gene family has been studied in several plants including *Arabidopsis* [19], *Oryza* [38], eggplant [39], grapevine [40], populus [41], brassica [42], and *Zea mays* [38]. However, the information about this crucial gene family in pineapple is still obscure. Due to the great significance of CBL-CIPK genes in various physiological and developmental processes, it is of great interest to investigate the CBL-CIPK genes in pineapple. Genome sequence of pineapple [43] provides an opportunity for genome-wide investigation of the CBL-CIPK genes. In this study, we identified eight CBL and 21 CIPK genes and performed a comprehensive analysis including exon-intron organization, chromosome distribution, and phylogenetic analysis. Additionally, we have performed expression analysis to identify the involvement of these genes in different biological and developmental stages in pineapple. We further characterized *Ac*CBL1 by overexpressing it in *Arabidopsis*. Our study provides a basis for further utilization of CBL-CIPK genes to develop next-generation crops especially in the context of a globally changing environment.

## 2. Materials and Methods

### 2.1. Identification of CBL-CIPK Genes in Pineapple Genome

The CBL and CIPK gene sequences from *Arabidopsis* and *Oryza sativa* were downloaded from TAIR (http://www.arabidopsis.org) and China Rice Data Center (http://www.ricedata.cn/gene/index.htm) respectively. Further, the CBL and CIPK genes were used as keywords against the pineapple genome database of phytozome (https://phytozome.jgi.doe.gov/pz/portal.html) to identify CBL-CIPK genes from pineapple. Additionally, Hidden Markov Model (HMM) profile of the NAF domain (PF03822) from the Pfam database (http://pfam.xfam.org/) and EF-hand calcium-binding domain (PS50222) from prosite (https://prosite.expasy.org/) were downloaded. We then performed searches from the pineapple genome using the HMM profiles by BLAST-P with the e-value set at 0.01. The identified sequences were further verified and redundant sequences were removed from the list. Finally, ExPASy (http://web.expasy.org/compute_pi/) was used determine the isoelectric point (pI) and molecular weight (MW) of CBL and CIPK proteins of pineapple.

### 2.2. Phylogenetic Analysis

The phylogenetic relationship of CBL-CIPK proteins between pineapple, *Arabidopsis*, and rice were studied using identified CBL-CIPK amino acid sequences from pineapple, *Arabidopsis*, and rice. The multiple sequence alignments were performed by using MUSCLE 3.7 and the phylogenetic trees were constructed by MEGA7 using the Maximum likelihood (ML) method with default parameters with the bootstrap option *n* = 1000.

### 2.3. Gene Structure Analysis and Conserved Motif Identification

The schematic *Ac*CBL-*Ac*CIPK genes structures were drawn by the Gene Structure Display Server 130 (http://gsds.cbi.pku.edu.cn/). The conserved motifs in the pineapple *Ac*CBL-*Ac*CIPK proteins were identified by MEME program (http://meme.nbcr. net/meme/cgi-bin/meme.cgi).

### 2.4. Chromosome Location of CBL and CIPK Genes in Pineapple

The information about the location of *Ac*CBL and *Ac*CIPK genes on chromosomes were collected from phytozome. MapChart software was used to visualize the *Ac*CBL and *Ac*CIPK genes mapped on chromosomes based on the gene start position and the length of related chromosomes.

### 2.5. Plant Materials and Growth Condition and Treatments

#### 2.5.1. Pineapple Growth and Treatments

Two-month-old tissue culture raised pineapple (*Ananas comosus*) variety MD2 plants were obtained from Qin lab (http://www.qinlab.net) [44] and grown in plastic pots containing soil mix (peat moss:perlite = 2:1 (*v*/*v*)) placed at 30 °C with 16 h light/8 h dark photoperiod under an intensity of 70 μmol m^−2^ s^−1^ 70% humidity as described earlier by Chen et al. [45]. For the various stress treatment experiments, the pineapple plants were exposed to different treatments for 24, 48, and 72 h. The treatments included salinity stress (150 mM NaCl), osmotic stress (350 mM mannitol), heat (45 °C), and cold stress (4 °C) respectively. The leaf and root tissues from whole plants were harvested and frozen immediately in liquid nitrogen and stored at −80 °C until used.

#### 2.5.2. Arabidopsis Treatments and Root Growth Assay

The wild-type *Arabidopsis thaliana* (L.) Heynh (Col-0; CS60000) was obtained from the Arabidopsis Biological Resource Center (Columbus, OH, USA). All plants used were Columbian in background. Surface sterilized *Arabidopsis* seeds were placed in a round, 9 cm Petri plates on modified Hoagland’s medium [46,47] containing 1% (*w*/*v*) sucrose and 1% (*w*/*v*) agar. The plates were kept at 4 °C in the dark for 2 day for seed stratification. After stratification, the plates were transferred to a growth room at 22 °C with 16 h light/8 h dark photoperiod under an intensity of 100 μmol m^−2^ sec^−1^ and seedlings were grown vertically.

Salinity stress treatment and growth recovery were performed from T3 generation of 3 different *Ac*CBL1 overexpressing lines. 5-day-old seedlings were transferred to new plates containing the modified Hoagland medium supplemented with 150 mM NaCl and kept 10 days in a growth room. Primary roots were analyzed after 2 days, 5 days, and 10 days of transfer. The experiments were repeated at least three times with 8–10 seedlings per treatment. To measure salinity induced root growth inhibition seedlings were photographed by digital camera (NIKON D750, Japan) and root growth was analyzed by ImageJ software (http://rsbweb.nih.gov/ij/).

For in-situ detection of hydrogen peroxide induced against biotic stress after exposure to fungus (*Sclerotinia sclerotiorum*) mature *Arabidopsis* rosette leaves were exposed to fungus for 24 h followed by staining with 3,3-diaminobenzidine (DAB) as reported earlier by Daudi et al. [48].

### 2.6. RNA Isolation and Quantitative Real-Time PCR (qRT-PCR) Analysis

Total RNA from desired tissues was isolated by using the RNeasy kit (Qiagen, MD, USA), followed by treatment with DNase I (Thermo Fisher Scientific, CA, USA) and first-strand cDNA synthesis by reverse transcription using the ThermoScript RT-PCR kit (Thermo Fisher Scientific, CA, USA). Quantitative PCR was performed by using FastStart DNA Master SYBR Green I master mix (Takara, Shiga, Japan) in a CFX96 qPCR system (Bio-Rad, Singapore). Primers used in qRT-PCR are listed in supplementary Table S3. The PCR cycle conditions for quantitative PCR were as follows: 95 °C for 2 min followed by 40 cycles of 95 °C for 10 s, 60 °C for 15 s, and 72 °C for 15 s. For each analysis, two technical replicates from three biological replicates were performed and pineapple EF1a was used as an internal control to normalize the mRNA levels, and finally, the fold change of genes was calculated using the 2^−ΔΔCT^ method.

### 2.7. RNA-Seq and Data Analysis

RNA was extracted from different developmental stages as described earlier by Chen et al., [45], pineapple of MD2 variety through Plant RNeasy Mini Kit (Qiagen MD, USA). Illumina sequencing was performed as previously described [45], with 1 μg RNA per sample and two independent biological replicates per genotype. cDNA libraries were constructed using the NEBNext Ultra™ RNA Library Prep Kit for Illumina (NEB, MA, USA) following standard protocols. The transcript abundance of pineapple CBL-CIPK genes were calculated as fragments per kilobase of exon model per million mapped reads (FPKM). The heatmap was created by pheatmap packages in R based the log2 (FPKM + 1).

### 2.8. Vector Constructs

*Ac*CBL1:GFP was generated by amplifying 678 bp of coding sequence without the stop codon from pineapple cDNA using the primers listed in Table S3. The amplified PCR fragment was then cloned into the pENTR/D-TOPO vector (Invitrogen, Carlsbad, CA, USA). pENTR/D-TOPO clones were then recombined into the destination vector pGWB505 by using LR Clonase II (Invitrogen, Carlsbad, CA, USA). Before performing the bacterial and plant transformation, the construct was confirmed by sequencing. 

### 2.9. Live Cell Imaging

For live cell microscopy, five-day-old *Ac*CBL1 overexpressing plants were treated either by 150 mM salt or wounded manually. After the treatment, the seedlings were mounted in the water on a cover glass for observation on a TCS SP8 microscope (Leica, Wetzlar, Germany) and imaged.

### 2.10. Statistical Analysis

Results are expressed as the means ± standard error (SE) from at least 3 experiments. To analyze statistical significance, a two-tailed Student’s *t*-test was used. 

## 3. Results

### 3.1. Identification of the CBL-CIPK Genes in Pineapple

A total of 8 CBL and 26 CIPK genes were initially obtained from the pineapple genome using BLAST, HMM search, and available pineapple annotation. Five redundant CIPK genes were removed after the Pfam NAF domain (PF03822) investigation identified putative CIPK genes. Finally, a total of 8 CBLs and 21 CIPK gene models were selected and annotated based on their gene ID in ascending order (Table 1a,b). All the CBLs contained the EF-hand and CIPKs possess the conserved NAF/FISL motif. The detailed information of the identified CBL-CIPK genes such as gene name, gene ID, protein length, isoelectric points, and molecular weights is listed in Table 1. Among 8 identified CBLs, *Ac*CBL6 was the smallest having 193 amino acid and *Ac*CBL4 was largest with 359 amino acid, whereas *Ac*CIPK8 was smallest with 382 amino acids and *Ac*CIPK20 with 506 amino acid was largest in the group. The molecular weight of the *Ac*CBL proteins ranged from 21.9 kDa to 40.8 kDa and 43.7 kDa to 55.8 kDa for CIPK proteins.

### 3.2. Gene Structure and Phylogenetic Analysis of Pineapple CBL-CIPK Genes

**Figure 1:**
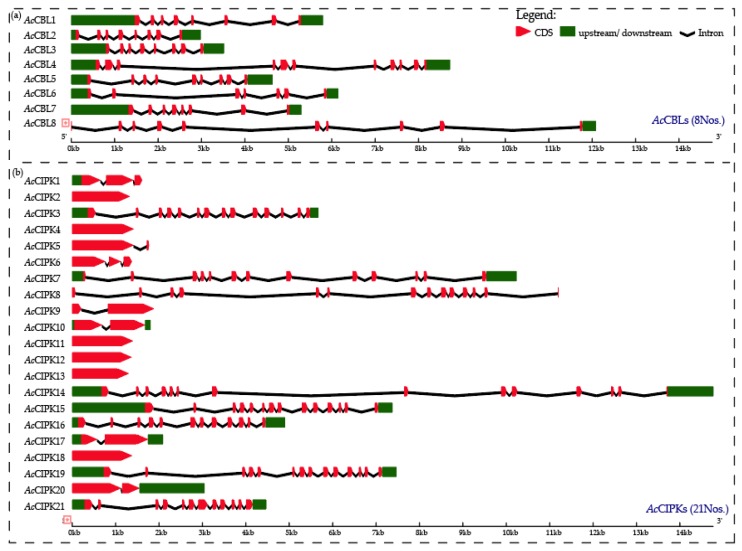
Exon-intron structure of pineapple CBL-CIPK genes. (**a**) Exon-intron structure of pineapple CBL genes (**b**) exon-intron structure of pineapple CIPK genes; green boxes indicate untranslated 5′- and 3′-regions; red boxes indicate exons; black lines indicate introns. Prefix ‘Ac’ indicates *Ananas comosus*.

The evolutionary aspect and structural diversity of the CBL-CIPK genes in pineapple were explored by studying the exon-intron organization. The exon-intron structure for *Ac*CBLs and *Ac*CIPKs were constructed using the online Gene Structure Display Server 2.0 [49]. All the *Ac*CBLs possessed introns which ranged from six to ten in numbers (*Ac*CBL6 had six introns, *Ac*CBL1, *Ac*CBL2, *Ac*CBL3, and *Ac*CBL7 possessed seven introns, *Ac*CBL5 had eight introns, *Ac*CBL8 had nine introns, and *Ac*CBL4 possessed maximum ten introns) (Figure 1a). The result also showed that all the *Ac*CBLs contained the UTR, though *Ac*CBL8 had only 3’ UTR. The distribution of introns in *Ac*CIPK genes ranged from zero to thirteen where *Ac*CIPK2, *Ac*CIPK4, *Ac*CIPK11, *Ac*CIPK12, *Ac*CIPK13, and *Ac*CIPK18 did not possess introns. *Ac*CIPK5, *Ac*CIPK9, *Ac*CIPK10, *Ac*CIPK17, and *Ac*CIPK19 possessed single intron, *Ac*CIPK1 and *Ac*CIPK6 had two introns, *Ac*CIPK21 had eleven introns, *Ac*CIPK16, and *Ac*CIPK7 had 12 introns, *Ac*CIPK3, *Ac*CIPK8, *Ac*CIPK14, *Ac*CIPK15, and *Ac*CIPK19 possessed maximum thirteen introns (Figure 1b). Moreover, no UTRs were observed in ten *Ac*CIPKs (*Ac*CIPK2, *Ac*CIPK4, *Ac*CIPK5, *Ac*CIPK6, *Ac*CIPK8, *Ac*CIPK9, *Ac*CIPK11, *Ac*CIPK12, *Ac*CIPK13, and *Ac*CIPK18) whereas two *Ac*CIPKs (*Ac*CIPK1 and *Ac*CIPK20) possessed only one UTR (5’ UTR and 3’ UTR respectively). Additionally, pineapple CBL-CIPK protein structures were further analyzed by performing MEME analysis. Overall, ten conserved motifs were identified in both *Ac*CBL and *Ac*CIPK protein (Supplementary Figures S1 and S2).

**Figure 2:**
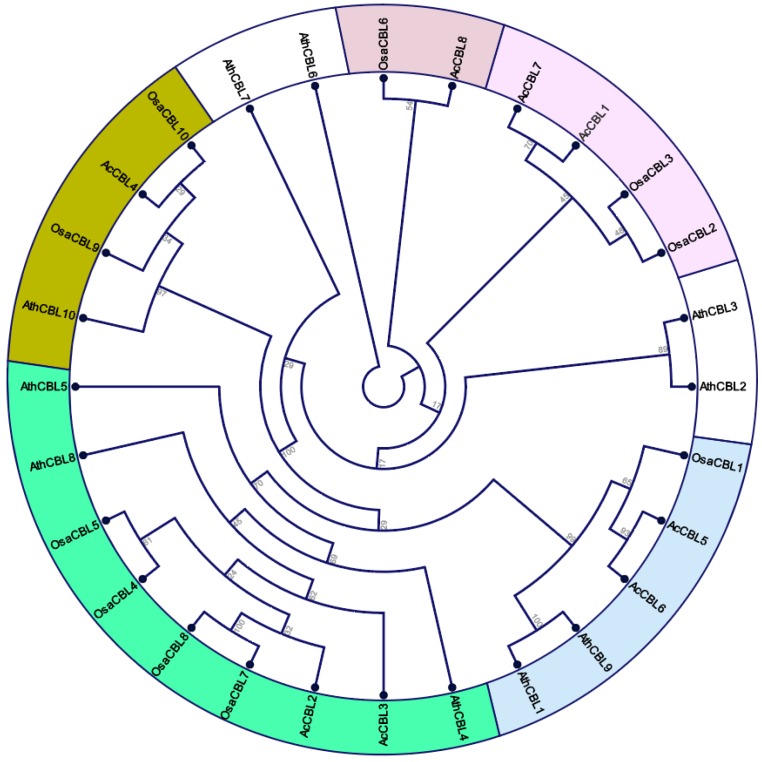
Phylogenetic tree depicting the relationships among CBL proteins from pineapple, rice, and *Arabidopsis*. The different colored arcs indicate different subgroups. Prefix ‘Ath’, Osa, and ‘Ac’ indicate CBL proteins from *Arabidopsis*, *Oryza sativa*, and *Ananas comosus* respectively.

**Figure 3:**
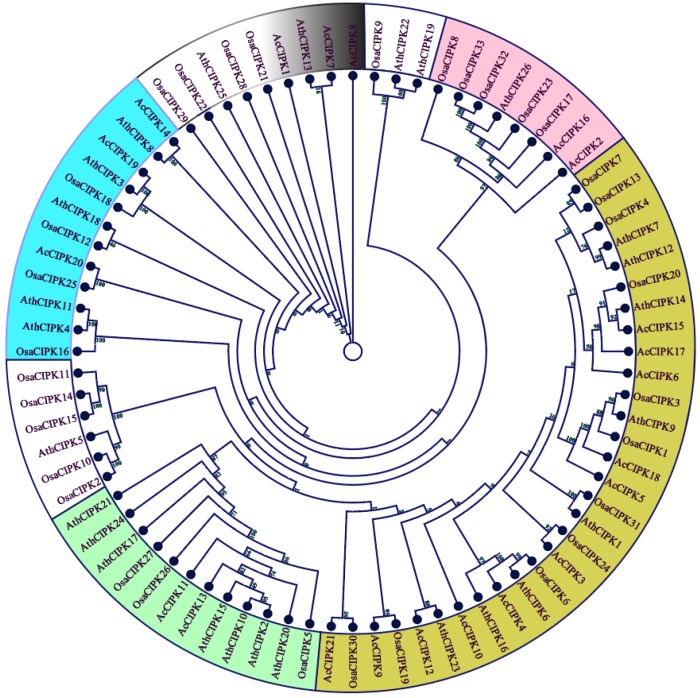
Phylogenetic tree depicting the relationships among CIPK proteins from pineapple, rice, and *Arabidopsis*. The different colored arcs indicate different subgroups. Prefix ‘Ath’, Osa, and ‘Ac’ indicate CBL proteins from *Arabidopsis*, *Oryza sativa*, and *Ananas comosus* respectively.

The evolutionary relationships and functional associations of CBL-CIPK genes in pineapple were investigated by constructing a multi-species phylogenetic tree using full-length protein sequences of CBL (Figure 2) and CIPK (Figure 3) from pineapple, *Arabidopsis*, and rice using the Maximum Likelihood method. *Ac*CBLs in phylogenetic trees formed four subfamilies more closely related to rice compared to *Arabidopsis*. The tree also divided the *Ac*CBLs into 5 subfamilies viz. *Ac*CBL1 and *Ac*CBL7 formed a group with *Osa*CBL2 and *Osa*CBL3, *Ac*CBL5 and *Ac*CBL6 formed a group with *Osa*CBL1, and *Ac*CBL6 paired with *Osa*CBL6 (Figure 2). Interestingly, some groups (depicted without any color in Figure 2 and Figure 3) both among CBL and CIPK did not have any members from pineapple, indicating the evolutionary divergence between monocotyledonous and dicotyledonous plants species.

### 3.3. Chromosomal Distribution of Pineapple CBL-CIPK Genes

**Figure 4:**
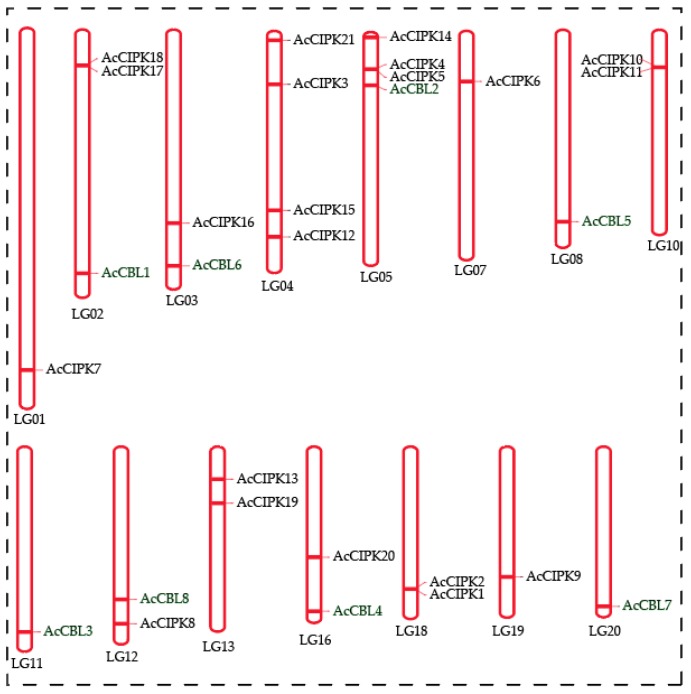
Chromosomal locations of pineapple CBL and CIPK genes. The 8 CBL and 21CIPK genes of pineapple were mapped to different chromosomes using MapChart.

The pineapple CBL-CIPK genes were unevenly distributed on 15 linkage groups (LG) out of 25 linkage groups present in the genome. The linkage groups LG 06, 09, 14, 15, 17, 21, 22, 23, 24, and 25 did not contain any CBL or CIPK genes (Figure 4). All 8 CBLs were located on different linkage groups, whereas CIPKs were located on different linkage groups ranging from 1-4 in number. Linkage groups LG01, 03, 07, 12, 16, and 19 had a single CIPK gene, LG 02, 08, 13, and 18 had two CIPK genes, LG05 had 3 CIPK genes and maximum 4 CIPK genes were located on LG 04 (Figure 4). The result showed no correlation between the length of linkage groups and number of CBL-CIPK gene distribution.

### 3.4. Expression Profiling of Pineapple CBL-CIPK Genes in Different Developmental Stages

**Figure 5:**
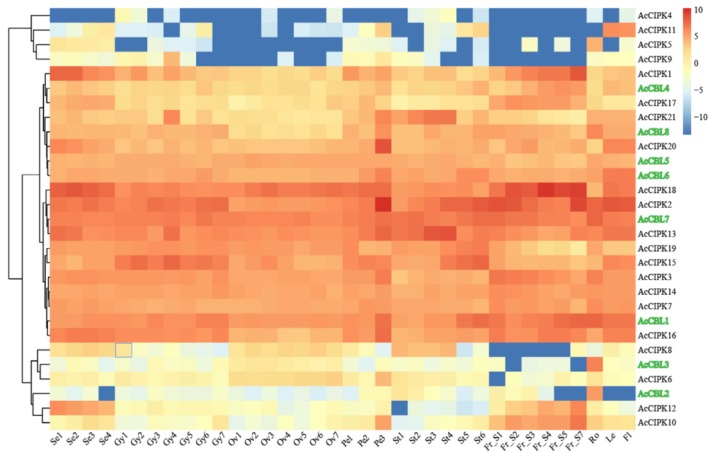
Expression profiles of the pineapple CBL and CIPK genes. Hierarchical clustering of expression profiles of pineapple CBL-CIPK genes in different organs and developmental stages. Red color indicates high levels of transcript abundance, and blue color indicates low transcript abundance. The color scale is shown at right side of the figure. Sample details are mentioned at the bottom of each lane: sepal Se1–Se4, gynoecium Gy1- Gy7, ovule Ov1–Ov7, petal Pe1–Pe3, stamen St1–St6, fruit ‘Fr_S1–Fr_S7’, root ‘Ro’, leaf ‘Le’, and flower ‘Fl’. Where ‘S’ is the abbreviation for ‘stage’. Pineapple CBL gene names are represented in green color.

The expression profiling of all the CBL-CIPK genes was carried out from RNA-Seq data generated from different organs of the pineapple plant. The expression profiles of all the pineapple CBL-CIPK genes were generated and hierarchically clustered using average log values of each gene (Figure 5). The hierarchical clustering of RNA-Seq data divided them into three major groups. In group I, low expressed genes (*Ac*CIPK4, *Ac*CIPK5, *Ac*CIPK9, and AcCIPK11) were clustered together. In contrast, group II contained genes (6 *Ac*CBLs and 13 *Ac*CIPK genes) with high level of expression in all the selected stages (Figure 5). Whereas, group III had genes (2 *Ac*CBLs and 4 *Ac*CIPK genes) with relatively low levels of expression. The expression pattern of CBL-CIPK genes suggests that they are expressed differentially during different developmental stages and contribute essentially to plant growth and development.

### 3.5. Expression Profiling of Pineapple CBL1 Gene in Response to Different Treatments

**Figure 6:**
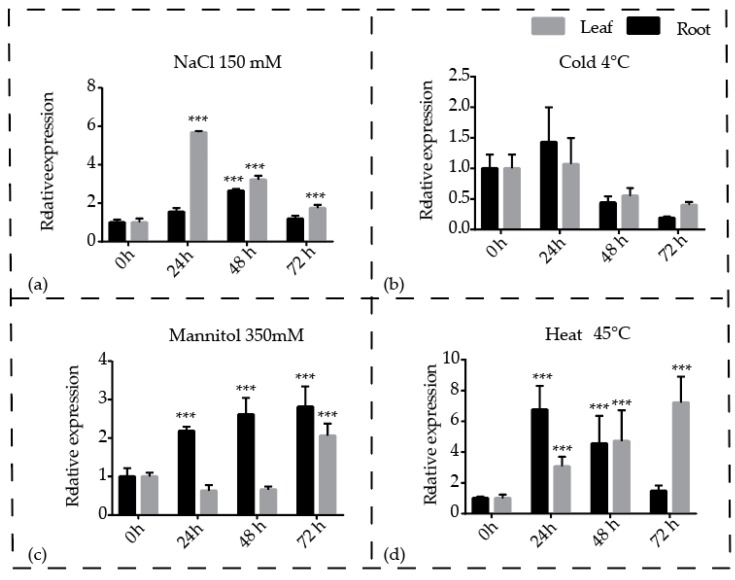
Expression profiles of the pineapple CBL1 gene in response to different stress treatments. qRT-PCR of CBL1 gene in at different time point (0 h, 24 h, 48 h, and 72 h) and samples (leaf and root) after (**a**) salt (NaCl 150 mM), (**b**) cold (4 °C) stress, (**c**) osmotic (Mnnitol 350 mM) stress, and (**d**) heat (45 °C) stress. Data were normalized to EF1a gene. Vertical bars indicate SE and *** indicates significantly different values compared to control treatment (*p* < 0.001). All experiments were performed with three technical and three biological repeats.

In the RNA-Seq experiment *Ac*CBL1 gene showed the highest expression, so we selected it for further study and investigated the role of *Ac*CBL1 gene during abiotic stress response. For this, we used quantitative real-time PCR (qRT-PCR) and determined the spatial and temporal expression profile of *Ac*CBL1 in different tissues (root and leaf) of pineapple at different time intervals (0, 24, 48, and 72 h) after salinity stress (150 mM NaCl), osmotic stress (350 mM mannitol), heat (45 °C), and cold stress (4 °C) treatments (Figure 6). The qPCR data showed that compared to untreated plants the transcript level of *Ac*CBL1 significantly increased during salinity, osmotic, and heat treatments whereas, the cold stress did not show significant response. The results also showed the differential expression of *Ac*CBL1 when it was compared between root and leaf (Figure 6). Collectively, these results strongly suggest that the *Ac*CBL1 gene contributes to various abiotic stresses in pineapple.

### 3.6. Salt Stress Increases the Localization of AcCBL1 in Arabidopsis Roots

**Figure 7:**
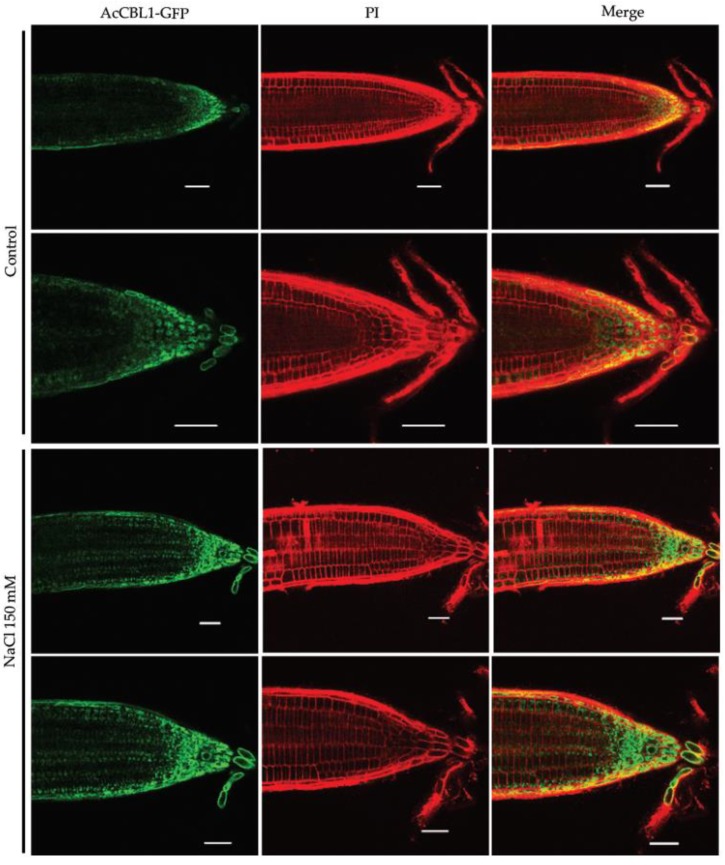
*Ac*CBL1-GFP localizes in cytosol under salt stress. Confocal analyses show *Ac*CBL1-GFP localization in the cytosol when of 5-day-old seedlings grown in the standard conditions were transferred in a medium containing 150 mM NaCl. GFP fluorescence is represented in green and propidium iodide (PI)fluorescence channel is represented in red. Scale bars = 50 μm.

**Figure 8:**
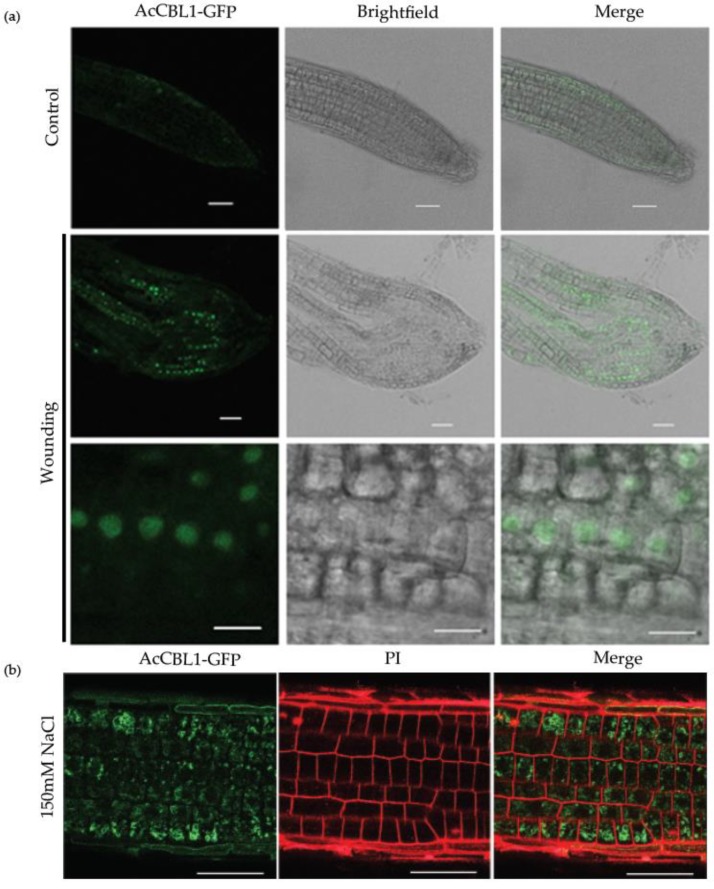
*Ac*CBL1-GFP translocalizes to nucleus from cytosol after injury. (**a**) Localization *Ac*CBL1-GFP in the nucleus when the 7-day-old root of transgenic seedlings is injured. (**b**) *Ac*CBL1-GFP localization in cytosol after salt stress. GFP fluorescence is represented in green and PI fluorescence channel is represented in red. Scale bars = 50 μm.

Having established the role of *Ac*CBL1 in salinity, osmotic, and heat stress in pineapple. We further investigated the biological function of *Ac*CBL1 by ectopically expressing *Ac*CBL1 gene in *Arabidopsis* under CaMV 35S promoter and GFP tag. Using a confocal microscope we monitored the localization of *Ac*CBL1:GFP after transfer experiment. Transfer experiment was carried out with five-day-old seedlings by transferring them to a fresh plate consisting of Hoagland medium supplemented with 150 mM NaCl for 48 h. The results showed that the *Ac*CBL1:GFP mainly localized in the cytosol and its expression increased after salt stress treatment (Figure 7 and Figure 8b). The increased localization of *Ac*CBL1 in *Arabidopsis* roots clearly indicates its involvement in salt stress. Taken together, our results indicate that the *Ac*CBL1 helps the plant to thrive well under salt stress.

### 3.7. Injury Induces AcCBL1 Translocation from Cytosol to the Nucleus

Previous studies have established that *Arabidopsis* CBLs are induced during wounding and injury [50]. In order to investigate the possible role of *Ac*CBL1 during injury, we monitored the expression of *Ac*CBL1:GFP after manually injuring the *Ac*CBL1 overexpressing plants. Interestingly, we found that the expression of *Ac*CBL1 was regulated by wounding and *Ac*CBL1 was translocated to the nucleus after the injury (Figure 8a). The results clearly indicate that the *Ac*CBL1 is regulated by injury and may be involved in the protection against injury stress.

### 3.8. Overexpression of AcCBL1 Results in Resistance to Salinity, Osmotic, and Biotic Stress

**Figure 9:**
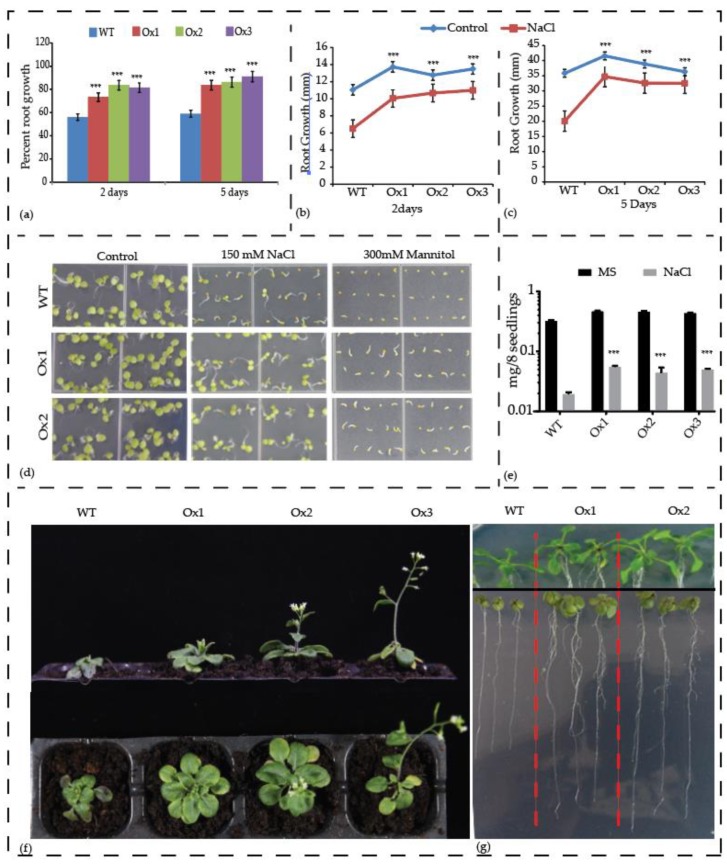
Overexpression of *Ac*CBL1 results in resistance to salinity and osmotic stress. (**a**) Percent root growth in 5-day-old wild-type and *Ac*CBL1 overexpressing seedlings transferred to new plates supplemented with 150 mm NaCl and analyzed after 2 days and 5 days of transfer. (**b**) Primary root growth after 2 days and (**c**) primary root growth after 5 days and (**d**) phenotype of 7-day-old *Ac*CBL1 overexpressing seedlings germinated under salinity (150 mM NaCl) and osmotic stress (300 mM mannitol). (**e**) Fresh weight of 5-day-old wild-type and *Ac*CBL1 overexpressing lines grown for an additional 10 days on 150 mM NaCl after transfer to new plate and analysis. (**f**) 15-day-old seedlings were subjected to saline irrigation with 250 mM NaCl for an additional 10 days and then photographed. (**g**) Seedlings grown for 5 days and transferred to new plate with (lower panel), or without (upper panel), 150 mM NaCl for 10 additional days and photographed. Vertical bars represent ± S.E., and *** indicates significantly different values between treatments (*p* < 0.001).

**Figure 10:**
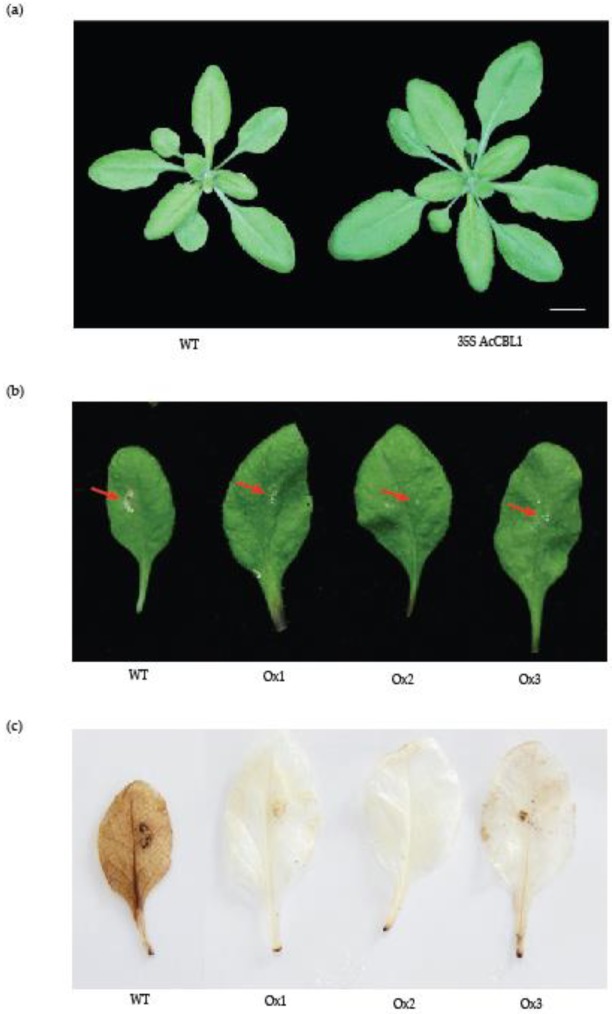
Overexpression of *Ac*CBL1 results in resistance to biotic stress. (**a**) Morphology of four-week-old wild-type (Col-0) and *Ac*CBL1 overexpressing plant. (**b**) Damage in leaves by exposure to fungus (*Sclerotinia sclerotiorum*) to mature rosette leaves of wild-type and 3 different lines (Ox1, Ox2, and Ox3) of *Ac*CBL1 overexpressing plants for 24 h. (**c**) Detection of H_2_O_2_ by DAB staining in leaves of wild-type (Col-0) and *Ac*CBL1 overexpressing lines after 24 h infection of fungus (*Sclerotinia sclerotiorum*). The brown color reflects the reactive oxygen species (ROS) accumulation after the fungus treatment. Scale bar = 1 cm.

As shown in Figure 6 and Figure 7, the expression of *Ac*CBL1 is significantly increased during osmotic and salinity stress, suggesting that this gene may be participating in abiotic stress responses including salinity stress. Previously, the involvement of *At*CBL1 and *At*CBL10 in response to salt stress has been well documented [17,35,51]. To test whether *Ac*CBL1 contributes to salinity stress we took different approaches: (1) We performed seedling transfer experiment of *Ac*CBL1 overexpressing lines and observed the salinity effect on primary root growth and percent root growth compared to wild-type plants; (2) tested the germination and growth phenotype under continuous salt and osmotic stress; (3) checked the performance of *Ac*CBL1 overexpressing lines under saline irrigation and (4) performed the fresh weight analysis of seedling after 10 days (d) of transfer experiment. In agreement to our hypothesis, *Ac*CBL1 overexpressing *Arabidopsis* lines showed better growth performance on 150 mM NaCl containing plates when compared to wild-type plants in terms of percent root growth (Figure 9a), and primary root growth (Figure 9b,c), after 2 d and 5 d of the transfer. The *Ac*CBL1 overexpressing lines also showed better germination rates under salt and osmotic (300 mM mannitol) stress (Figure 9d). Consistently, fresh weights of the seedling grown on medium containing NaCl were significantly more compared to wild type plants (Figure 9e,g). Moreover, the saline irrigation of 15 d old plants with 250 mM NaCl for 10 d also showed better growth performance compared to wild-type (Figure 9f). Additionally, to investigate the contribution of *Ac*CBL1 overexpressing lines to resistance to biotic stresses, we infected the mature rosette leaves with plant pathogenic fungus (*Sclerotinia sclerotiorum*) for 24 h and performed DAB staining. The DAB staining results indicated that the *Ac*CBL1 lines had less accumulation and distribution of hydrogen peroxide (Figure 10b), indicating the increased tolerance of these plants under biotic stress. Collectively, the above results convincingly show that the *Ac*CBL1 contributes to abiotic and biotic stress and *Ac*CBL1 overexpressing plants display better performance under unfavorable stress conditions.

## 4. Discussion

Due to their sessile nature, plants face several biotic and abiotic stresses during their life cycle. Several genes contribute in defense and protective measure for plants to thrive well under hostile conditions [37,52,53]. CBL-CIPK gene family consists of plant-specific and calcium involving signaling modules indispensable to various stress signaling pathways. Due to availability of sequenced genome, CBL-CIPK gene families have been extensively studied in several plant species [19,38,39,40,41,42,54] however, the information about pineapple CBL-CIPK genes remain elusive. In the present study, a comprehensive search of CBL-CIPK genes from pineapple genome resulted in the identification of eight CBL and 21 CIPK genes, which were designated as *Ac*CBL1 to *Ac*CBL8 and *Ac*CIPK1 to *Ac*CIPK21 on the basis of their gene ID in the order of increasing number. 

In plants, the CBL-CIPK network represents an excellent module for decoding Ca^2+^ signals during stress. This network is also a classic example of a diverged Ca^2+^ signal decoding system for sensing differential Ca^2+^ signals in numerous stress signaling pathways [42]. CBL proteins interact with different CIPK proteins depending on the cellular Ca^2+^ signals (concentration) that determine the precise outcome due to specific complex formation [55]. A 24 amino acid domain, referred to as NAF domain (due to the presence of conserved Asn-Ala-Phe), is specific and sufficient to mediate the interaction between CBL and CIPK [18]. We also utilized NAF domain as a query to identify CIPK proteins in pineapple genome. 

Gene structure (intron-exon structures) in the gene families are often considered as imprints of evolution [56]. During the genome duplication, the deletion and insertion or both could happen to the genes. The intron–exon structure of the identified *Ac*CBL genes showed that the CDS of all the *Ac*CBLs were discontinuous by the presence of introns (Figure 1a). Whereas, *Ac*CIPK genes can be grouped in intron less or intron poor and intron rich genes (Figure 1b). The intron less or intron-poor and intron-rich genes of CIPKs are conserved among other genomes such as *Arabidopsis*, maize, grapevine, and *Brassica* [19,38,40,42]. The intron numbers of *Ac*CBL and *Ac*CIPK genes ranged from 0 to 13 showing the large differences between the structures of the paralogs. This difference could be one of the attributes of functionality difference among the paralogs of CBL-CIPK genes. The phylogenetic analysis serves as an excellent method to analyze evolutionary relationships among genes [57]. In the present study, phylogenetic analysis of *Ac*CBL and *Ac*CIPK genes, together with *Arabidopsis* and rice, categorized *Ac*CBLs mainly into five different groups (Figure 2) and CIPKs also into five different groups (Figure 3). Consistent with the current information of plant evolution, in the phylogenetic tree *Ac*CBLs were more closely related to rice CBLs (monocot) compared to *Arabidopsis* (dicot). These findings indicate that they may have arisen together by gene duplication via a common ancestor and could have similar functions. It is interesting to note that the lower plants contain a lesser number of CBL-CIPK genes; whereas their number remarkably increases in higher land plants. The abundance of CBL-CIPK genes could be due to the increase in the complexity of the land plants during their evolution [58]. We found that the pineapple genome contains eight CBL genes (Table 1a) compared to 10 CBLs of *Arabidopsis* and rice whereas, 21 CIPK genes (Table 1b) compared to 26 CIPKs in *Arabidopsis* and 34 CIPKs in rice. The multiple CBL-CIPK genes present in the genome of the pineapple indicates the significance of the CBL-CIPK gene family in growth, development, and response to various stimuli [6].

Considering the importance of the CBL-CIPK genes in overall plant growth and response, we studied their expression profile in different tissues and development stages of pineapple. The expression profile suggests that *Ac*CBL and *Ac*CIPK genes express differentially in different tissues and stages (Figure 5). The hierarchical clustering of the gene expression data grouped them into three different categories (Figure 5). RNA-Seq data from different tissues and stages clearly indicated that the CBL-CIPK genes are involved in the regulation of different biological functions correlated to tissue or stage. For example, *Ac*CIPK18 was highly expressed during the different stages of pineapple fruit development, suggesting that it may be involved in the regulation of pineapple fruit development (Figure 5).

Increasing evidence suggests that the CBL-CIPK toolkit performs a plethora of functions in plant growth and development under optimal and stress conditions [6,20,21,22,59]. In our study, we found that both the *Ac*CBL1 and *Ac*CBL7 were highly expressed in RNA-seq (Figure 5). Therefore, we selected *Ac*CBL1 to further investigate its expression under different stress conditions. Real-time quantitative RT-PCR analysis showed that the transcript level of *Ac*CBL1 significantly changed during all the stress treatments applied. The *Ac*CBL1 exhibited significant changes in root and leaf at all the time points under salt stress, osmotic (mannitol), cold, and heat stress (Figure 6). The real-time result indicates that *Ac*CBL1 could be playing a key role in adaption to extreme conditions as *Arabidopsis* CBL1 contributes to salt, cold, and osmotic stress [29]. Several studies indicate that CBLs contribute to salt stress [6,29,35]. In *Arabidopsis*, *At*CBL4 with CIPK24, along with Na+/H+ antiporter SOS1, function in exclusion of Na^+^ from the cytoplasm [3,6]. To further validate the hypothesis, we generated transgenic *Arabidopsis* plants overexpressing *Ac*CBL1 tagged with GFP. Consistently, the overexpression lines also show the increased localization of *Ac*CBL1 in roots under salt stress (Figure 7 and Figure 8b). Previously, *At*CBL1 was reported to be induced after wounding [16,54]. Consistent with these reports, we also found that *Ac*CBL1 is associated with wounding. *Ac*CBL1 protein was induced and translocated to the nucleus after wounding (Figure 8a). Moreover, the independent homozygous T3 transgenic lines (designated as Ox1, Ox2, Ox3) showed better performance under salt stress in terms of present root growth (Figure 9a), primary root growth (Figure 9b,c), germination (Figure 9d), fresh weight (Figure 9e), and overall growth phenotype (Figure 9f,g). 

Additionally, fungal infection followed by DAB (3,3-diaminobenzidine) staining resulted in the better performance of *Ac*CBL1 overexpressing transgenic plants compared to wild-type plants. In the presence of peroxidases, DAB is oxidized to generate a dark brown precipitate which is easily monitored to detect the existence and dispersal of hydrogen peroxide in cells [48]. The biotic stress would result in the generation of reactive oxygen species (ROS), resulting in oxidative stress to plants. After 24 h of fungal stress the transgenic plants displayed less damage to leaves (Figure 10b) and less accumulation of hydrogen peroxide (Figure 10c) than wild-type. In plants, the elevation in the cytosolic calcium (Ca^2+^) has been previously documented [60]. The *Ac*CBL1 could be sensing the change in the cytosol after salt and wound stress, and relaying the signal downstream would be helping the *Ac*CBL1 overexpressing plant to perform better under stress conditions.

Taken together, our results clearly indicate that ectopic expression of *Ac*CBL1 enhances salt and biotic stress tolerance by facilitating scavenging of the Na^+^ and ROS from the cell.

## 5. Conclusions

In the present study, a comprehensive analysis of CBL-CIPK gene family was carried out in the economically important pineapple plant. A total of 8 CBL and 21 CIPK proteins were identified and characterized. The expression profile of pineapple CBL-CIPK genes suggest that *Ac*CBL and *Ac*CIPK proteins play key roles in the pineapple growth, development, and response to abiotic stresses. Further, the functional characterization of *Ac*CBL1 shed light on its biological role and contribution during salt stress and wounding. The present study could be a foundation for further research and serve as a basis to utilize CBL-CIPK genes for development of next-generation crops.

## Figures and Tables

**Table 1:** Characteristics of calcineurin B-like (CBL) and CBL-interacting protein kinases (CIPK) genes identified from pineapple. Prefix ‘Ac’ Indicates *Ananas comosus*.

(a) Characteristics of Pineapple CBL Gene					
Name	Transcript ID	Chromosome Location	Length	MW	pI
*Ac*CBL1	Aco000875	LG02:16012605-16018403	226	25,958.74	4.81
*Ac*CBL2	Aco004447	LG05:3255687-3258668	213	24,478.92	4.81
*Ac*CBL3	Aco005668	LG11:12099528-12103047	216	24,888.39	4.86
*Ac*CBL4	Aco005877	LG16:10709202-10717927	359	40,867.51	4.68
*Ac*CBL5	Aco011645	LG08:12541062-12545693	234	26,797.47	4.72
*Ac*CBL6	Aco012888	LG03:15510233-15516379	193	21,928.83	4.52
*Ac*CBL7	Aco015301	LG20:10385809-10391109	224	25,763.52	4.81
*Ac*CBL8	Aco024403	LG12:9915463-9927546	252	28,939.19	6.16
**(b) Characteristics of Pineapple CIPK Genes**					
*Ac*CIPK1	Aco001621	LG18:9219002-9220612	409	43,926.73	9.35
*Ac*CIPK2	Aco001625	LG18:9192567-9193895	442	50,063.84	9.26
*Ac*CIPK3	Aco002215	LG04:3262495-3268163	450	50,565.19	8.78
*Ac*CIPK4	Aco004322	LG05:2160825-2162243	472	52,996.59	6.69
*Ac*CIPK5	Aco004323	LG05:2169056-2170823	487	54,823.09	8.75
*Ac*CIPK6	Aco005225	LG07:3071294-3072667	405	42,654.61	9.99
*Ac*CIPK7	Aco006700	LG01:22600010-22610245	385	44,053.77	7.12
*Ac*CIPK8	Aco007507	LG12:11547144-11558347	382	43,701.04	6.87
*Ac*CIPK9	Aco008201	LG19:8409312-8411200	424	45,918.02	8.61
*Ac*CIPK10	Aco010015	LG10:2187779-2189581	478	54,071.92	6.51
*Ac*CIPK11	Aco010017	LG10:2198749-2200149	466	52,913.08	9.06
*Ac*CIPK12	Aco011132	LG04:13486171-13487538	455	49,351.15	6.94
*Ac*CIPK13	Aco012533	LG13:1862440-1863744	434	49,113.68	9.01
*Ac*CIPK14	Aco014260	LG05:45295-60064	422	47,747.21	7.71
*Ac*CIPK15	Aco015008	LG04:11703020-11710396	449	50,875.82	8.01
*Ac*CIPK16	Aco015525	LG03:12649272-12654172	396	45,072.72	8.99
*Ac*CIPK17	Aco016931	LG02:2099383-2101473	451	50,452.21	8.09
*Ac*CIPK18	Aco016932	LG02:2036912-2038291	459	50,213.68	9.08
*Ac*CIPK19	Aco019253	LG13:3471010-3478477	441	50,378.76	7.2
*Ac*CIPK20	Aco021653	LG16:7092847-7095892	506	55,836.96	6.63
*Ac*CIPK21	Aco022003	LG04:312075-316545	444	49,867.09	6.87

## Supplementary Materials

The following are available online at https://www.mdpi.com/2218-273X/9/7/293/s1, Figure S1: The distribution of conserved motifs in pineapple CBL proteins, Figure S2: The distribution of conserved motifs in pineapple CIPK proteins, Table S3: List of primers used in present study.

## Abbreviations

CBL	Calcineurin B-like
CIPK	CBL-interacting protein kinases
Ca^2+^	Calcium ion
ER	Endoplasmic reticulum
CaM	Calmodulin
CDPKs	Calcium-dependent protein kinases
SOS	Salt overlay sensitive
UTR	Untranslated region
CDS	Coding sequence
MEME	Multiple Em for Motif Elicitation
LG	Linkage groups
DAB	3,3-diaminobenzidine

## References

[1] Dodd A.N., Kudla J., Sanders D. (2010). The language of calcium signaling. Annu. Rev. Plant Biol..

[2] Tang R.J., Luan S. (2017). Regulation of calcium and magnesium homeostasis in plants: From transporters to signaling network. Curr. Opin. Plant Biol..

[3] Zhu J.K. (2016). Abiotic Stress Signaling and Responses in Plants. Cell.

[4] Costa A., Navazio L., Szabo I. (2018). The contribution of organelles to plant intracellular Calcium signalling. J. Exp. Bot..

[5] Tuteja N., Mahajan S. (2007). Calcium signaling network in plants: An overview. Plant Signal. Behav..

[6] Kudla J., Becker D., Grill E., Hedrich R., Hippler M., Kummer U., Parniske M., Romeis T., Schumacher K. (2018). Advances and current challenges in calcium signaling. New Phytol..

[7] Ding Y., Shi Y., Yang S. (2019). Advances and challenges in uncovering cold tolerance regulatory mechanisms in plants. New Phytol..

[8] Behera S., Zhaolong X., Luoni L., Bonza M.C., Doccula F.G., De Michelis M.I., Morris R.J., Schwarzlander M., Costa A. (2018). Cellular Ca(2+) Signals Generate Defined pH Signatures in Plants. Plant Cell..

[9] Meena M.K., Prajapati R., Krishna D., Divakaran K., Pandey Y., Reichelt M., Mathew M.K., Boland W., Mithofer A., Vadassery J. (2019). The Ca2+ Channel CNGC19 Regulates Arabidopsis defense against Spodoptera Herbivory. Plant Cell..

[10] Van Loon L.C. (2016). The Intelligent Behavior of Plants. Trend. Plant Sci..

[11] De Vriese K., Himschoot E., Dünser K., Nguyen L., Drozdzecki A., Costa A., Nowack M.K., Kleine-Vehn J., Audenaert D., Beeckman T. (2019). Identification of Novel Inhibitors of Auxin-Induced Ca^2+^ Signaling via a Plant-Based Chemical Screen. Plant Physiol..

[12] Blatt M.R., Grabov A. (1997). Signal redundancy, gates and integration in the control of ion channels for stomatal movement. J. Exp. Bot..

[13] Michard E., Simon A.A., Tavares B., Wudick M.M., Feijo J.A. (2017). Signaling with Ions: The Keystone for Apical Cell Growth and Morphogenesis in Pollen Tubes. Plant Physiol..

[14] Bender K.W., Zielinski R.E., Huber S.C. (2018). Revisiting paradigms of Ca^(2+)^ signaling protein kinase regulation in plants. Biochem. J..

[15] Ranty B., Aldon D., Cotelle V., Galaud J.P., Thuleau P., Mazars C. (2016). Calcium Sensors as Key Hubs in Plant Responses to Biotic and Abiotic Stresses. Front. Plant Sci..

[16] Kudla J., Xu Q., Harter K., Gruissem W., Luan S. (1999). Genes for calcineurin B-like proteins in Arabidopsis are differentially regulated by stress signals. Proc. Natl. Acad. Sci. USA.

[17] Liu J., Zhu J.K. (1998). A calcium sensor homolog required for plant salt tolerance. Science.

[18] Albrecht V., Ritz O., Linder S., Harter K., Kudla J. (2001). The NAF domain defines a novel protein-protein interaction module conserved in Ca2+-regulated kinases. EMBO J..

[19] Kolukisaoglu U., Weinl S., Blazevic D., Batistic O., Kudla J. (2004). Calcium sensors and their interacting protein kinases: Genomics of the Arabidopsis and rice CBL-CIPK signaling networks. Plant Physiol..

[20] Zhang Y., Lv Y., Jahan N., Chen G., Ren D., Guo L. (2018). Sensing of Abiotic Stress and Ionic Stress Responses in Plants. Int. J. Mol. Sci..

[21] Xuan Y.H., Kumar V., Han X., Kim S.H., Jeong J.H., Kim C.M., Gao Y., Han C.D. (2019). CBL-INTERACTING PROTEIN KINASE 9 regulates ammonium-dependent root growth downstream of IDD10 in rice (*Oryza sativa*). Ann. Bot..

[22] Yang Y., Wu Y., Ma L., Yang Z., Dong Q., Li Q., Ni X., Kudla J., Song C.-P., Guo Y. (2019). The Ca2+ sensor SCaBP3/CBL7 Fine Tunes Arabidopsis Alkali Tolerance and Modulats Plasma Membrane H+-ATPase Activity. Plant Cell.

[23] Li Z.Y., Xu Z.S., He G.Y., Yang G.X., Chen M., Li L.C., Ma Y. (2013). The voltage-dependent anion channel 1 (AtVDAC1) negatively regulates plant cold responses during germination and seedling development in Arabidopsis and interacts with calcium sensor CBL1. Int. J. Mol. Sci..

[24] Huang C., Ding S., Zhang H., Du H., An L. (2011). CIPK7 is involved in cold response by interacting with CBL1 in Arabidopsis thaliana. Plant Sci..

[25] Ligaba-Osena A., Fei Z., Liu J., Xu Y., Shaff J., Lee S.C., Luan S., Kudla J., Kochian L., Pineros M. (2017). Loss-of-function mutation of the calcium sensor CBL1 increases aluminum sensitivity in Arabidopsis. New Phytol..

[26] Lan W.Z., Lee S.C., Che Y.F., Jiang Y.Q., Luan S. (2011). Mechanistic analysis of AKT1 regulation by the CBL-CIPK-PP2CA interactions. Mol. Plant.

[27] Li Z.Y., Xu Z.S., Chen Y., He G.Y., Yang G.X., Chen M., Li L.C., Ma Y.Z. (2013). A novel role for Arabidopsis CBL1 in affecting plant responses to glucose and gibberellin during germination and seedling development. PLoS ONE.

[28] Mahs A., Steinhorst L., Han J.P., Shen L.K., Wang Y., Kudla J. (2013). The calcineurin B-like Ca2+ sensors CBL1 and CBL9 function in pollen germination and pollen tube growth in Arabidopsis. Mol. Plant.

[29] Cheong Y.H., Pandey G.K., Grant J.J., Batistic O., Li L., Kim B.G., Lee S.C., Kudla J., Luan S. (2007). Two calcineurin B-like calcium sensors, interacting with protein kinase CIPK23, regulate leaf transpiration and root potassium uptake in Arabidopsis. Plant J..

[30] Eckert C., Offenborn J.N., Heinz T., Armarego-Marriott T., Schultke S., Zhang C., Hillmer S., Heilmann M., Schumacher K., Bock R. (2014). The vacuolar calcium sensors CBL2 and CBL3 affect seed size and embryonic development in Arabidopsis thaliana. Plant J..

[31] Liu L.L., Ren H.M., Chen L.Q., Wang Y., Wu W.H. (2013). A protein kinase, calcineurin B-like protein-interacting protein Kinase9, interacts with calcium sensor calcineurin B-like Protein3 and regulates potassium homeostasis under low-potassium stress in Arabidopsis. Plant Physiol..

[32] Held K., Pascaud F., Eckert C., Gajdanowicz P., Hashimoto K., Corratge-Faillie C., Offenborn J.N., Lacombe B., Dreyer I., Thibaud J.B. (2011). Calcium-dependent modulation and plasma membrane targeting of the AKT2 potassium channel by the CBL4/CIPK6 calcium sensor/protein kinase complex. Cell Res..

[33] Yang Y., Guo Y. (2018). Elucidating the molecular mechanisms mediating plant salt-stress responses. New Phytol..

[34] Lin H., Yang Y., Quan R., Mendoza I., Wu Y., Du W., Zhao S., Schumaker K.S., Pardo J.M., Guo Y. (2009). Phosphorylation of SOS3-LIKE CALCIUM BINDING PROTEIN8 by SOS2 protein kinase stabilizes their protein complex and regulates salt tolerance in Arabidopsis. Plant Cell..

[35] Kim B.G., Waadt R., Cheong Y.H., Pandey G.K., Dominguez-Solis J.R., Schultke S., Lee S.C., Kudla J., Luan S. (2007). The calcium sensor CBL10 mediates salt tolerance by regulating ion homeostasis in Arabidopsis. Plant J..

[36] Zhang J., Liu J., Ming R. (2014). Genomic analyses of the CAM plant pineapple. J. Exp. Bot..

[37] Xie T., Chen C., Li C., Liu J., Liu C., He Y. (2018). Genome-wide investigation of WRKY gene family in pineapple: Evolution and expression profiles during development and stress. BMC Genom..

[38] Chen X., Gu Z., Xin D., Hao L., Liu C., Huang J., Ma B., Zhang H. (2011). Identification and characterization of putative CIPK genes in maize. J. Genet. Genom..

[39] Li J., Jiang M.M., Ren L., Liu Y., Chen H.Y. (2016). Identification and characterization of CBL and CIPK gene families in eggplant (Solanum melongena L.). Mol. Genet. Genom..

[40] Xi Y., Liu J., Dong C., Cheng Z.M. (2017). The CBL and CIPK Gene Family in Grapevine (Vitis vinifera): Genome-Wide Analysis and Expression Profiles in Response to Various Abiotic Stresses. Front. Plant Sci..

[41] Zhang H., Yin W., Xia X. (2008). Calcineurin B-Like family in Populus: Comparative genome analysis and expression pattern under cold, drought and salt stress treatment. Plant Growth Regul..

[42] Yin X., Wang Q., Chen Q., Xiang N., Yang Y., Yang Y. (2017). Genome-Wide Identification and Functional Analysis of the Calcineurin B-like Protein and Calcineurin B-like Protein-Interacting Protein Kinase Gene Families in Turnip (Brassica rapa var. rapa). Front. Plant Sci..

[43] Ming R., Wai C.M., Guyot R. (2016). Pineapple Genome: A Reference for Monocots and CAM Photosynthesis. Trends Genet..

[44] Priyadarshani S., Hu B., Li W., Ali H., Jia H., Zhao L., Ojolo S.P., Azam S.M., Xiong J., Yan M. (2018). Simple protoplast isolation system for gene expression and protein interaction studies in pineapple (*Ananas comosus* L.). Plant Method..

[45] Chen P., Li Y., Zhao L., Hou Z., Yan M., Hu B., Liu Y., Azam S.M., Zhang Z., Rahman Z.U. (2017). Genome-Wide Identification and Expression Profiling of ATP-Binding Cassette (ABC) Transporter Gene Family in Pineapple (*Ananas comosus* (L.) Merr.) Reveal the Role of AcABCG38 in Pollen Development. Front. Plant Sci..

[46] Baskin T.I., Wilson J.E. (1997). Inhibitors of protein kinases and phosphatases alter root morphology and disorganize cortical microtubules. Plant Physiol..

[47] Rahman A. (2013). Auxin: A regulator of cold stress response. Physiol. Plant.

[48] Daudi A., O’Brien J.A. (2012). Detection of Hydrogen Peroxide by DAB Staining in Arabidopsis Leaves. Bio Protoc..

[49] Hu B., Jin J., Guo A.Y., Zhang H., Luo J., Gao G. (2015). GSDS 2.0: An upgraded gene feature visualization server. Bioinformatics.

[50] Yadav A.K., Jha S.K., Sanyal S.K., Luan S., Pandey G.K. (2018). Arabidopsis calcineurin B-like proteins differentially regulate phosphorylation activity of CBL-interacting protein kinase 9. Biochem. J..

[51] Manik S.M., Shi S., Mao J., Dong L., Su Y., Wang Q., Liu H. (2015). The Calcium Sensor CBL-CIPK Is Involved in Plant’s Response to Abiotic Stresses. Int. J. Genom..

[52] Liu C., Xie T., Chen C., Luan A., Long J., Li C., Ding Y., He Y. (2017). Genome-wide organization and expression profiling of the R2R3-MYB transcription factor family in pineapple (Ananas comosus). BMC Genom..

[53] Tak H., Negi S., Ganapathi T.R. (2017). Banana NAC transcription factor MusaNAC042 is positively associated with drought and salinity tolerance. Protoplasma.

[54] Niu L., Dong B., Song Z., Meng D., Fu Y. (2018). Genome-Wide Identification and Characterization of CIPK Family and Analysis Responses to Various Stresses in Apple (*Malus domestica*). Int. J. Mol. Sci..

[55] Zhu J.K. (2003). Regulation of ion homeostasis under salt stress. Curr. Opin. Plant Biol..

[56] Boudet N., Aubourg S., Toffano-Nioche C., Kreis M., Lecharny A. (2001). Evolution of intron/exon structure of DEAD helicase family genes in Arabidopsis, Caenorhabditis, and Drosophila. Genome Res..

[57] Tamura K., Peterson D., Peterson N., Stecher G., Nei M., Kumar S. (2011). MEGA5: Molecular evolutionary genetics analysis using maximum likelihood, evolutionary distance, and maximum parsimony methods. Mol. Biol. Evol..

[58] Edel K.H., Marchadier E., Brownlee C., Kudla J., Hetherington A.M. (2017). The Evolution of Calcium-Based Signalling in Plants. Curr. Biol..

[59] Lyzenga W.J., Sullivan V., Liu H., Stone S.L. (2017). The Kinase Activity of Calcineurin B-like Interacting Protein Kinase 26 (CIPK26) Influences Its Own Stability and that of the ABA-regulated Ubiquitin Ligase, Keep on Going (KEG). Front. Plant Sci..

[60] Mousavi S.A., Chauvin A., Pascaud F., Kellenberger S., Farmer E.E. (2013). GLUTAMATE RECEPTOR-LIKE genes mediate leaf-to-leaf wound signalling. Nature.

