# Supplementary material for: Genome-Wide Identification and Expression Profiling of CBL-CIPK Gene Family in Pineapple (Ananas comosus) and the Role of AcCBL1 in Abiotic and Biotic Stress Response
Source: Biomolecules. 2019 Jul 20;9(7):293. doi: 10.3390/biom9070293 (PMC6681290; doi:10.3390/biom9070293)
Supplement: Supplementary file 1 [file biomolecules-09-00293-s001.zip › Supplementary material/Supplemenatry Table S3- Plrmer List .docx]

**Supplementary Table S3**. List of primers used in present study.

| Sl.No. | Name | Primer Seq 5-->3 | Remark |
| --- | --- | --- | --- |
| 1 | AcCBL1 F | caccATGTTGCAGTGCCTAGAGG | Used for Cloning |
| 2 | AcCBL1 R | GGTATCGTTGACTTGAGAGT |  |
| 3 | AcCBL1 F | GACAGGGAAGAGTGGCGAAAT | Used for qPCR |
| 4 | AcCBL1 R | AGTGGAACACAAAGCTTGGGA |  |
| 5 | AcEF1a F | TCTTCTCAGGGAAGGTCTCTAC |  |
| 6 | AcEF1a R | CTCTGCACACTCTTCACATACA |  |
