# Supplementary material for: Genome-Wide Identification and Expression Profiling of CBL-CIPK Gene Family in Pineapple (Ananas comosus) and the Role of AcCBL1 in Abiotic and Biotic Stress Response
Source: Biomolecules. 2019 Jul 20;9(7):293. doi: 10.3390/biom9070293 (PMC6681290; doi:10.3390/biom9070293)
Supplement: Supplementary file 1 [file biomolecules-09-00293-s001.zip › Supplementary material/Supplementary Figure .docx]

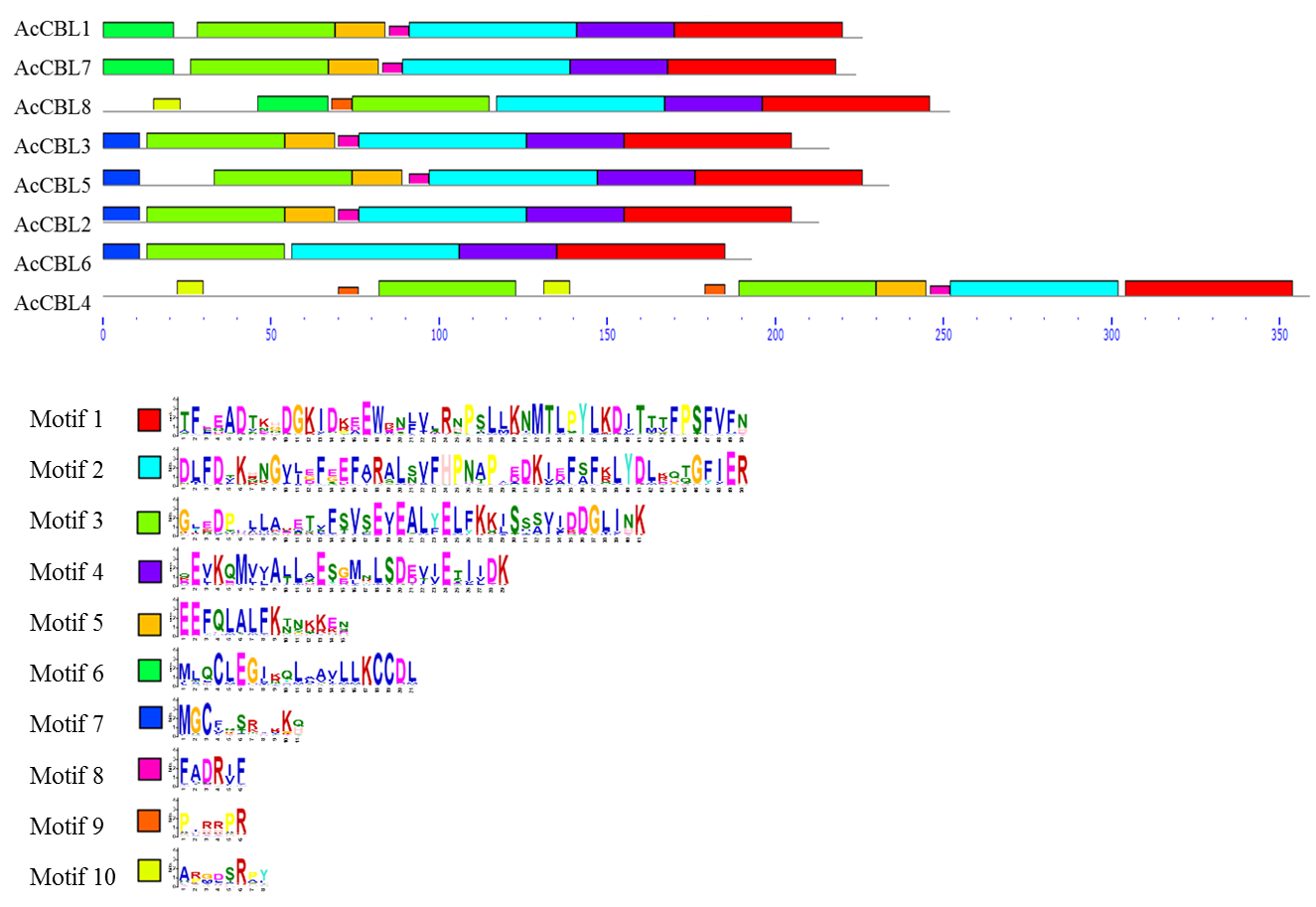


**Supplementary Figure S1.** The distribution of conserved motifs in pineapple CBL proteins.


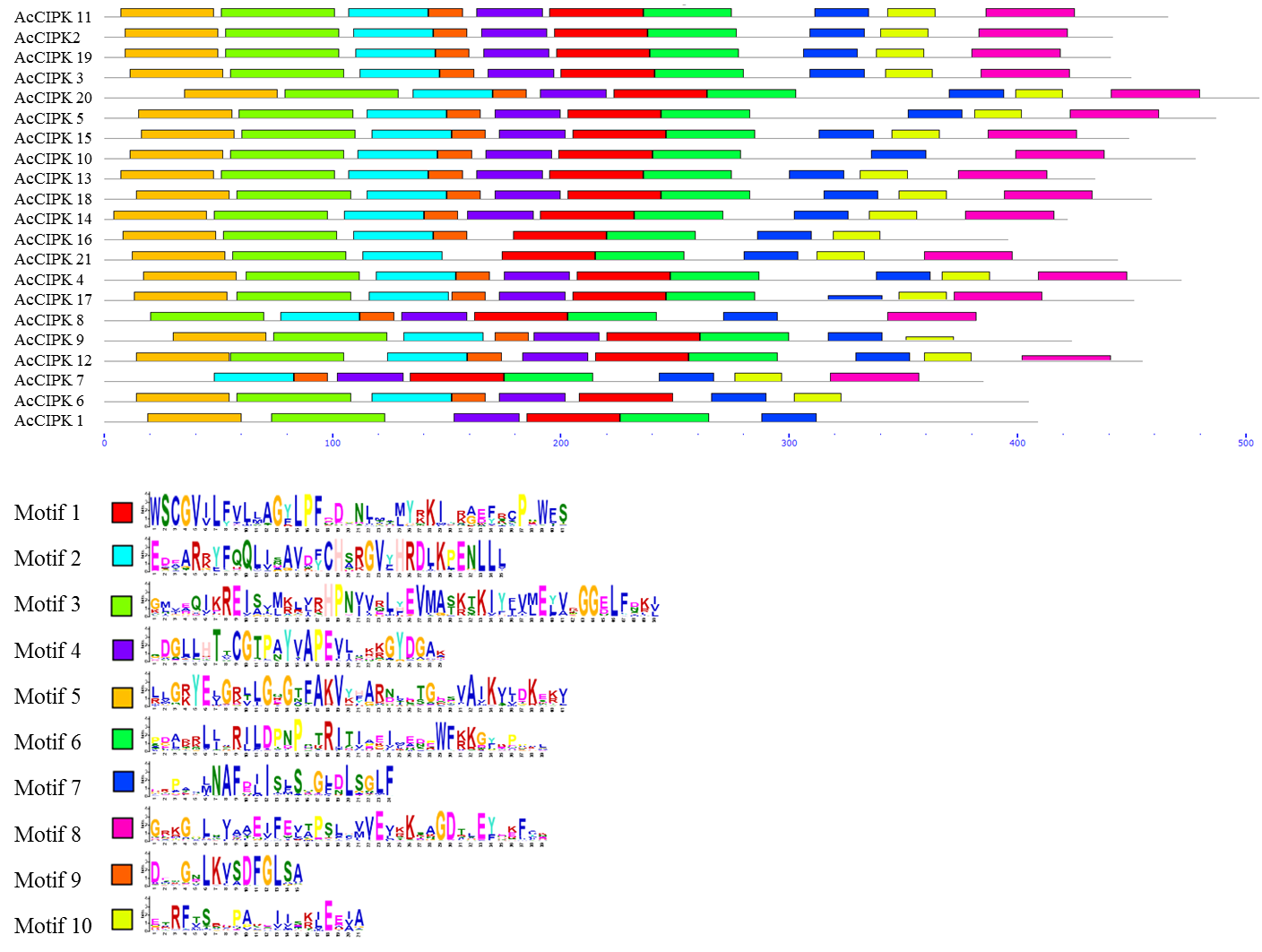


**Supplementary Figure S2.** The distribution of conserved motifs in pineapple CIPK proteins.
